# Steroid hormone regulation of immune responses in cancer

**DOI:** 10.1097/IN9.0000000000000012

**Published:** 2022-10-31

**Authors:** Ana C. Anderson, Nandini Acharya

**Affiliations:** 1 Evergrande Center for Immunologic Diseases, Ann Romney Center for Neurologic Diseases, Harvard Medical School and Mass General Brigham, Boston, MA, USA; 2 Pelotonia Institute for Immuno-Oncology, OSUCCC–James, The Ohio State University, Columbus, OH, USA; 3 Department of Neurology, The Ohio State University, Wexner Medical Center, Columbus, OH, USA

**Keywords:** glucocorticoids, androgens, cancer, immunotherapy, sex bias, T cell dysfunction/exhaustion

## Abstract

Steroid hormones are derived from cholesterol and can be classified into sex hormones (estrogens, androgens, progesterone) that are primarily synthesized in the gonads and adrenal hormones (glucocorticoids and mineralocorticoids) that are primarily synthesized in the adrenal gland. Although, it has long been known that steroid hormones have potent effects on the immune system, recent studies have led to renewed interest in their role in regulating anti-tumor immunity. Extra-glandular cells, such as epithelial cells and immune cells, have been shown to synthesize glucocorticoids and thereby modulate immune responses in the tumor microenvironment. Additionally, new insight into the role of androgens on immune cell responses have shed light on mechanisms underpinning the observed sex bias in cancer survival outcomes. Here, we review the role of steroid hormones, specifically glucocorticoids and androgens, in regulating anti-tumor immunity and discuss how their modulation could pave the way for designing novel therapeutic strategies to improve anti-tumor immune responses.

## 1. Introduction

The nervous, endocrine, and immune systems communicate closely to ensure optimal function of the immune system. Two neuroendocrine axes drive the production of steroid hormones (adrenal and sex hormones) known to have potent effects on immune responses. The hypothalamic–pituitary–adrenal (HPA) axis is the major source of the adrenal hormone, glucocorticoid (GC). The main activator of the HPA axis is the neuropeptide corticotropin-releasing hormone (CRH), synthetized in the hypothalamic paraventricular nucleus (PVN). When CRH reaches the anterior pituitary, it stimulates corticotroph cells to synthesize and release of adrenocorticotropic hormone (ACTH) ^[1,2]^. ACTH in turn is secreted into the blood circulation from where it reaches the adrenal cortex to stimulate the synthesis and secretion of mainly GCs and to a lesser extent mineralocorticoids and adrenal androgens ^[3]^. Importantly, the HPA axis can also be activated by infections and inflammatory processes. Indeed, the production of GCs by the HPA axis in response to inflammation is important for resolving inflammation and restoring immune homeostasis. The hypothalamic–pituitary–gonadal (HPG) axis controls the production of sex hormones. Neurons in the hypothalamus produce and secrete gonadotropin releasing hormone (GnRH). GnRH binds to receptors on gonadotroph cells in the anterior pituitary to stimulate their production of luteinizing hormone (LH) and follicle-stimulating hormone (FSH), which stimulate gonadal production of the sex hormones androgen (testosterone), estrogen, and progesterone. The HPA and the HPG axes are involved in intimate crosstalks with each other such that the function of one is affected by the activation of the other ^[4,5]^.

Steroid hormones have long been recognized to have important effects on the immune system ^[6–8]^. GCs were first used to suppress inflammation in patients with rheumatoid arthritis in the 1940s. Since then, synthetic GCs have been the standard treatment for reducing inflammation and immune activation in many inflammatory disorders, including asthma, allergic rhinitis, dermatological, ophthalmic, neurological and autoimmune diseases, allotransplantation, and sepsis ^[9,10]^. In cancer, GCs are currently the standard treatment for immune-related adverse events (irAEs), inflammatory reactions that develop in patients treated with immunotherapy ^[11]^. Sex hormones also have a role in cancer. It has long been recognized that there is sex bias in the immune response to cancer with females exhibiting more potent immune responses compared to males ^[12]^. In this review, we will discuss recent studies that have elucidated some of the mechanisms by which steroid hormones, specifically GCs and androgens, shape anti-tumor immune responses.

## 2. Steroid hormones: mechanisms of action

Steroid hormones are lipophilic molecules that bind to steroid hormone receptors (SHRs) that reside in the cytosol within target cells where they are complexed to chaperones, eg, heat shock protein 90 (Hsp90). Upon binding to their ligands, SHRs dissociate from their chaperones and undergo a structural change that exposes their nuclear localization signal. After translocation to the nucleus, SHRs can bind to hormone response elements (HREs) in the promoter regions and transactivate gene expression. Alternatively, SHRs can bind to other transcription factors and interfere with their activity, a process known as trans-repression. SHRs can thus modulate numerous responses in a large variety of cells, wherein their effects depend on the cellular context.

## 3. Steroid hormone regulation of immune responses in the tumor microenvironment

### 3. 1. Glucocorticoid

GCs play a critical role in shaping immune responses in the tumor microenvironment (TME). Through study of the transcriptome of tumor-infiltrating CD8^+^ T cell subsets, we demonstrated that *Nr3c1* (gene encoding glucocorticoid receptor; GR) is highly expressed by dysfunctional or exhausted CD8^+^ T cells ^[13]^, thus associating GR activity with suppressed CD8^+^ T cell responses. Although, GR-mediated suppression of inflammation in tumors had been previously ascribed to interference with activator protein 1 (AP1) and nuclear factor-κB (NFκB) activity ^[14,15]^, we showed that the GR bound to the promotor region and transactivated the expression of the immune checkpoint receptors PD1, Tim3, Lag3, Tigit, and the immune suppressive cytokines such as Interleukin (IL)-10 in CD8^+^ T cells. Unsupervised analysis of the genes induced by the GR in CD8^+^ T cells showed that the GR promoted the expression of the gene program associated with T cell dysfunction. Additionally, GC signaling could synergize with IL-27 signaling to further augment expression of the dysfunction gene program in CD8^+^ T cells in the TME. We also found that the GR had a role in precursor CD8^+^ T cells, where it promoted expression of T cell factor-1 (TCF1), a transcription factor that restrains effector differentiation ^[16,17]^ and plays a fundamental role in maintaining stemness in CD8^+^ T cells ^[18–21]^. Accordingly, we showed that loss of the GR in CD8^+^ T cells accelerated effector differentiation but prevented development of dysfunctional phenotype. Thus, the GC-GR axis acts at multiple points along the effector CD8^+^ T cell differentiation trajectory to shape anti-tumor CD8^+^ T cell responses. Importantly, we ^[13]^ and others ^[22]^ have demonstrated that GCs can be produced locally in the TME by myeloid cells and CD4^+^ T cells and that abrogating local GC production can reduce tumor burden in murine models of cancer.

GCs can also regulate the function of CD8^+^ T cells by modulating their metabolic state. Treatment of CD8^+^ T cells with GCs was shown to lead to long-lasting suppression of glycolysis and consequently impaired effector function, memory formation, and anti-tumor activity ^[23]^. In macrophages, GC treatment was shown to promote genes involved in the tricarboxylic acid (TCA) cycle but inhibit glycolysis by suppressing hypoxia-inducible factor 1-alpha (HIF1α); however, how this impacted anti-tumor CD8^+^ T cell responses was not investigated ^[24]^.

The impact of GC signaling on CD8^+^ T cell responses is important clinically. We showed that GC signature genes were more highly expressed in immune checkpoint blockade (ICB) non-responding than responding patients ^[13]^. Similarly, a recent study that identified transcription factors differentially active in ICB responders vs non-responders found deactivation of multiple transcription factors, including the GR and the androgen receptor (AR) in CD8^+^ T cells from ICB responders ^[25]^. Further, a study of chromatin accessible regions in primary tumor biopsies from patients with basal cell carcinoma (BCC) receiving anti-PD-1 immunotherapy showed that dysfunctional CD8^+^ T cells had increased chromatin accessibility at regions containing GR motifs ^[26]^. In line with the above observations, blockade of the GC-GR axis has been shown to enhance ICB efficacy in animal models ^[13]^. Indeed, it is plausible that differences in the magnitude of GC-GR signaling in different TMEs underlies the observed variation in ICB response rates across tumor types.

As noted above, patients that experience irAEs upon ICB are treated with GCs. Early studies indicated that administration of GCs did not affect the objective response rate (ORR) following ICB therapy ^[27]^. However, several recent studies have indicated that patients on ICB that receive GCs have lower response rates. One study demonstrated that simultaneous, but not subsequent, administration of corticosteroids with ICB reduced CD8^+^ T cell proliferation and impaired anti-tumor immune responses ^[28]^. Further, overall survival (OS) was lower in anti-CTLA-4 ICB-treated melanoma patients that received corticosteroids early during treatment. Reduced survival and time to treatment failure were noted in patients receiving high-dose GC for the treatment of irAEs compared to patients who received low-dose GCs ^[29]^. Further, poor response to anti-PD-L1 ICB has been associated with patients on baseline steroids ^[30]^. These observations underscore the need to understand the effects of low- vs high-dose administration of GCs and how these relate to the effects of endogenous GCs. Of note, the administration of high-dose GC, most commonly dexamethasone (DEX), is a standard of care treatment in glioblastoma (GBM) and is widely used during the entire course of the disease including pre- and postoperative management, and during chemotherapy and radiotherapy. The primary purpose is to reduce tumor-associated vasogenic edema and to prevent or treat increased intracranial pressure. However, the immune-suppression mediated by GCs may negatively affect the survival of GBM patients. Indeed, a meta-analysis of data from a total of 8752 patients with GBM showed that the use of steroids during radiotherapy was associated with reduced OS and progression-free survival (PFS) and, importantly, was identified as an independent prognostic factor for poor prognosis ^[31]^. Overall, these data indicate that the dose and duration of GC treatment should be carefully calibrated to achieve treatment goals and prevent possible steroid-associated complications.

**Figure 1.**
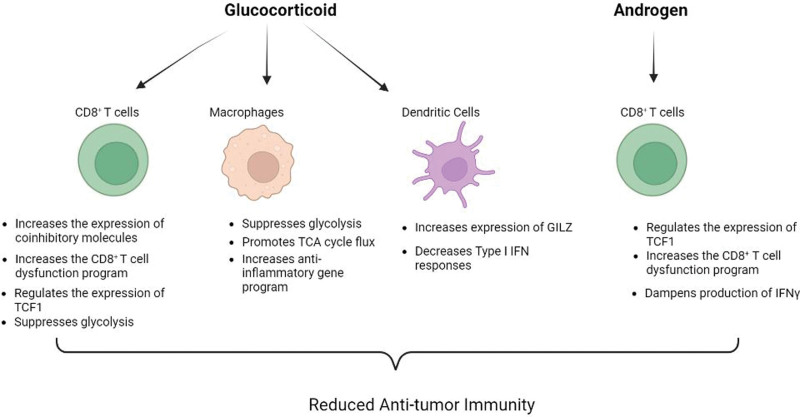
**Effects of steroid hormones on anti-tumor immune responses: Glucocorticoids and androgens affect the function and phenotype of different immune cell types in the tumor microenvironment and lead to attenuation of anti-tumor immune responses via the indicated mechanisms.** This figure was created with BioRender.com. IFN: interferon, TCF1: T cell factor-1, TCA: tricarboxylic acid.

Stress-induced production of GCs can also have a large impact on anti-tumor immunity. A study of the impact of psychological stress showed that systemic GC production curtailed anti-tumor immune responses by acting on dendritic cells (DCs) ^[32]^. Stress elevated the level of plasma GC leading to increased production of GC-inducible factor, glucocorticoid-induced leucine zipper (GILZ; encoded by TSC22D3), which abrogated type I interferon (IFN) responses in DC and decreased IFN-γ^+^T cells. Further, negative mood in patients with cancer showed a close correlation with plasma cortisol levels and TSC22D3 expression in circulating leukocytes. These results indicate that stress-induced GC can counter anti-tumor immunity. Thus, excessive GCs produced either systemically following psychological stress or locally in the TME can shape anti-tumor immunity (Figure 1), suggesting that blocking GC production or abrogating GR function can improve patient outcomes.

### 3. 2. Sex hormones

Males are more prone to developing cancer as compared to females ^[33]^. The sex bias is recapitulated in pre-clinical animal models where increased tumor burden has been observed in murine models of melanoma ^[34]^, colon cancer ^[35]^, GBM ^[36]^, and bladder cancer ^[37]^. Indeed, androgen ablation has been shown to boost the immune response and increase the efficacy of vaccination in a mouse model of prostate cancer ^[38]^. Although, underlying mechanisms have been unclear, a recent study revealed that CD8^+^ T cells from cancers in male subjects, including human patients and mice displayed more severe dysfunctional T cell phenotype ^[37]^. Sex differences in the growth pattern of murine bladder cancer (MB49) were eliminated in *Rag2* knockout mice suggesting that the observed sex bias was immune-mediated. This study demonstrated that androgen signaling favored the stem-like CD8^+^ T cell phenotype via promotion of the expression of TCF1, wherein AR acted as a direct transcriptional trans-activator of *Tcf*7 (gene encoding TCF1). Indeed, the AR was uniquely expressed in the stem-like CD8^+^ T cell subset and AR motif scanning identified four and five putative AR elements (AREs) within 1 kilobase of the promoter immediately upstream of the human and mouse *Tcf7* transcriptional start sites. Luciferase assays confirmed that AR could directly regulate *Tcf7* transcription. Loss of the AR resulted in decreased *T*cf7/TCF1 and accelerated effector differentiation. Accordingly, ablation of the androgen-AR axis rewired the TME to favor effector T cell differentiation and potentiate the efficacy of anti-PD-1 ICB. However, another recent study showed different effects of the AR on *Tcf7* ^[39]^. This study demonstrated reduced maintenance of stem-like CD8^+^ T cells and increased transition toward terminally exhausted CD8^+^ T cells in male mice relative to female mice in the MC38 and B16 tumor models. Female TCR transgenic CD8^+^ T cells had increased chromatin accessibility in the region upstream element of *Tcf7*, whereas their male counterparts had almost inaccessible chromatin at this region. Importantly, AR deletion in male TCR transgenic CD8^+^ T cells opened the chromatin region in the upstream element of *Tcf7*. By contrast, the *Tigit, Eomes, Lag3,* and *Havcr2* (gene encoding Tim3) genomic regions displayed increased chromatin accessibility in wild type male CD8^+^ T cells compared to female and male AR-deficient TCR transgenic CD8^+^ T cells. These data indicated that the AR suppressed the expression of TCF1 and enhanced the expression of genes involved in driving T cell dysfunction ^[18]^. In line with this, intra-tumoral CD8^+^ T cells from male patients displayed lower expression of stemness genes, such as *Sell, IL7r, Ccr7,* and *Tcf7*, along with higher expression of terminal exhaustion genes, including *Pdcd1, Havcr2, Tigit, Lag3, Tox,* and *Batf*, than the corresponding cells from female patients. Both of these studies attribute the sex bias in cancer to AR-mediated regulation of *Tcf7*/TCF1 in CD8^+^ T cells and the subsequent differentiation to dysfunctional T cells; however, the former ascribed a positive regulatory role while the latter a negative regulatory role. The disparity between the two studies could be due to the difference in the immunogenicity and thus trajectory of CD8^+^ T cell effector differentiation in the models studied and/or the abundance of environmental signals, such as type I IFN signaling that antagonizes TCF1 expression ^[40]^.

AR signaling can also limit the efficacy of immunotherapy. Advanced prostate cancer is refractory to ICB, however the clinical trial (NCT02312557) evaluating dual therapy with AR inhibition and anti-PD-1 ICB achieved a response rate of 18%, underscoring the importance of AR signaling axis in mediating resistance to ICB. Mechanistically, it was demonstrated that androgen response elements (AREs) were found within open chromatin regions next to *Ifng* where AR directly bound and repressed the *Ifng* expression. Further, AR blockade combined with androgen deprivation therapy (ADT) increased the T cell response to PD1 inhibition and prolonged survival in mouse models of prostate cancer and sarcoma ^[25]^. Similarly, a recent study demonstrated that anti-androgen therapy improved the efficacy of ICB therapy in pre-clinical models of bladder cancer ^[41]^. Collectively, these data establish the AR signaling pathway as a critical mechanism of curtailing both primary anti-tumor immune responses and responses to immunotherapy (Figure 1).

## 4. Steroidogenesis in immune cells

Not only do immune cells respond to steroid hormones but they can also produce these hormones ^[13,22,42–44]^, which can act in either autocrine or paracrine manner to shape immune responses. Steroid hormones are produced from the metabolic breakdown of cholesterol, which occurs primarily in the adrenal gland, gonads, and placenta. However, several studies have documented the presence of extra-glandular steroidogenesis in the brain ^[45,46]^, skin ^[47,48]^, thymus ^[49]^, adipose tissues ^[50,51]^, mucosa ^[52,53]^, and in immune cells ^[13,22,42–44]^. Human alveolar macrophages convert androstenedione to androgens, which in turn can regulate the phagocytic activity of these cells ^[54]^. Th2 cells can produce steroids in the context of parasitic worm infection and in the TME ^[22,42]^. We have shown previously that monocyte-macrophages lineage cells can produce steroids locally in the TME and augment T cell dysfunction ^[13]^. Moreover, we have recently shown that hyperactive unfolded protein response and redirected acetyl-coenzyme A increased steroidogenesis in DCs deficient for Bat3 in the context of both autoimmunity and cancer ^[55]^. The enhanced steroidogenesis in Bat3-deficient DC suppressed T cell responses resulting in the attenuation of autoimmunity and acceleration of tumor growth ^[55]^.

## 5. Conclusions

Steroid hormones play a fundamental role in shaping immune responses in cancer. Recent studies have elucidated some of the molecular mechanisms by which steroid hormones like GCs and androgen can affect CD8^+^ T cell function in the TME; however, detailed studies delineating the role of other steroid hormones such as mineralocorticoids, estrogens, and progesterone in modulating the function of CD8^+^ T cells in the TME are needed. It will be interesting to dissect out the potential antagonistic pathways by which male sex and female sex hormones regulate CD8^+^ T cell functions. Given that the molecular mechanisms by which the GR and AR regulate CD8^+^ T cell functions share features, it will be important to address whether the GR and AR, both of which are expressed in immune cells, function co-operatively or independently to determine functional phenotypes. SHRs are expressed by a wide array of immune cell populations in addition to CD8^+^ T cells, hence studies investigating their roles in regulating the function of different immune cell types will aid in generating a comprehensive understanding of the cellular circuitry utilized by steroid hormones to shape anti-tumor immune responses. As steroid hormones are produced from cholesterol, it will be interesting to connect how diet and cellular metabolism determine steroid hormone-mediated regulation of anti-tumor immunity. A deeper understanding of the mechanisms by which the GR, AR, estrogen receptor (ER), progesterone receptor (PR), and mineralocorticoid receptor (MR) affect immune cells will help the development of novel therapies employing immune cell type- and gender-specific perturbations for the treatment of cancer, as well as other diseases.

## Conflicts of interest

ACA is a member of the SAB for Tizona Therapeutics, Trishula Therapeutics, Compass Therapeutics, Zumutor Biologics, Excepgen, and ImmuneOncia, which have interests in cancer immunotherapy. ACA is also a paid consultant for iTeos Therapeutics and Larkspur Biosciences. ACA’s interests were reviewed and managed by the Brigham and Women’s Hospital and Partners Healthcare in accordance with their conflict of interest policies.

## Acknowledgments

Work in the author’s laboratory is supported by grants from the National Institutes of Health (R01CA229400). ACA is a recipient of the Brigham and Women’s Hospital President’s Scholar Award.
